# Tissue-resident memory T cells in the nasal mucosa: gatekeepers of chronicity and relapse in allergic rhinitis

**DOI:** 10.3389/fimmu.2026.1955001

**Published:** 2026-09-18

**Authors:** Xinyu Han, Fei Li, Qiqi Yang, Chunsong Liu, Yan Ruan, Weiping He, Chunqiao Li, Tengyu Chen, Baijing Huang, Gang Liu, Wenda Feng

**Affiliations:** 1 The First Affiliated Hospital of Guangzhou University of Chinese Medicine, Guangzhou, China; 2 ShenShan Hospital, The First Affiliated Hospital of Guangzhou University of Chinese Medicine, Shanwei, China; 3 Second Affiliated Hospital of Anhui University of Chinese Medicine, Hefei, China; 4 Department of Otolaryngology Head and Neck Surgery, The First Affiliated Hospital of Guangzhou University of Chinese Medicine, Guangzhou, China; 5 Lingnan Institute of Otolaryngology, Guangdong Clinical Research Academy of Chinese Medicine, Guangzhou, China; 6 Department of Otorhinolaryngology–Head and Neck Surgery, The First Affiliated Hospital of Anhui University of Chinese Medicine, Hefei, Anhui, China

**Keywords:** allergen immunotherapy, allergic rhinitis, chronicity, nasal mucosa, relapse, tissue-resident memory T cells, type 2 immunity

## Abstract

Allergic rhinitis (AR) is a pervasive type-2 inflammatory disorder of the nasal mucosa whose clinical course is defined less by acute flares than by recurrence and apparent resistance to cure. Although AR has traditionally been framed as a systemic, IgE-driven disease, a growing body of evidence points to the nasal mucosa itself as a site of durable, antigen-specific immune memory. Tissue-resident memory T (TRM) cells - non-recirculating, longevity-endowed lymphocytes that lodge in barrier tissues - are ideally positioned to convert transient allergen exposure into persistent inflammation. This mini review synthesizes human and preclinical evidence that nasal TRM cells, including Th2-polarized CD4+ subsets and a recently described pathogenic CD8+ subset (first characterized in recurrent chronic rhinosinusitis with nasal polyps, CRSwNP), constitute a credible - but still largely hypothetical - cellular substrate for AR chronicity and post-immunotherapy relapse. We propose that the 2025 discovery of granzyme K (GZMK)-expressing CD8+ memory T cells driving recurrent airway inflammation (Lan et al., Nature 2025) provides a translatable framework for understanding AR relapse. We note that direct, high-resolution human evidence for nasal TRM in pure AR remains limited; accordingly, we integrate findings from CRSwNP, lower-airway disease, and animal models as a translatable evidence base and explicitly flag where each conclusion is established versus hypothetical. We outline how targeting TRM retention, phenotype, or effector molecules could complement allergen immunotherapy.

## 1 Introduction

Allergic rhinitis affects up to 40% of the population and imposes a substantial burden through nasal obstruction, rhinorrhea, sneezing, and impaired quality of life (1). Current guidelines foreground pharmacotherapy and allergen immunotherapy (AIT), the only disease-modifying intervention (2, 3). Yet two clinical realities remain poorly explained: (i) symptoms recur season after season, and (ii) a substantial proportion of patients relapse after AIT is discontinued (2, 3). Both phenomena imply the persistence of a local, antigen-experienced cellular reservoir that standard systemic models do not capture.

The concept of immune memory in allergy has long concentrated on circulating IgE and central or effector memory T cells. However, the most abundant memory T-cell compartment in humans resides not in blood or lymphoid tissue but in non-lymphoid barrier tissues, where tissue-resident memory T (TRM) cells enforce rapid, local responses (4–8). In the nose - a chronically allergen-exposed mucosal surface - TRM cells may therefore be the missing link between exposure and recurrence, although direct demonstration in pure AR is still sparse. This review argues that nasal TRM cells are plausible gatekeepers of AR chronicity and relapse and should be a focal point for the next generation of mechanistic and therapeutic studies.

## 2 Nasal mucosal TRM cells: identity and phenotype

TRM cells are defined operationally by their persistence within non-lymphoid tissues and their failure to recirculate (9–11). In mice and humans they are marked by CD69, which antagonizes sphingosine-1-phosphate receptor 1 (S1PR1) and thereby retains cells in tissue (12, 13), and by adhesion molecules CD103 (aEb7, binding epithelial E-cadherin) and CD49a (VLA-1, binding collagen), which anchor them to epithelial and stromal structures (4). A core transcriptional program - driven by Hobit (Zfp683) and Blimp-1 (Prdm1) and reinforced by Runx3 - enforces residency and suppresses egress genes (9, 11, 14, 15). Operationally, CD69, CD103, CD49a and CXCR6 each indicate a TRM-associated phenotype, but no single marker establishes functional tissue residency on its own; residency is best inferred from their convergent expression together with direct demonstration of non-recirculation (9, 11). Compartmentalization studies show that human barrier tissues harbor substantial TRM pools distinct from peripheral blood (5). In the upper airway, nasal tissues - studied largely in CRSwNP - contain abundant TRM (16, 17), whereas the lower airway harbors its own resident CD4+ and CD8+ TRM pools (18–20).

Crucially, TRM are not a uniform population. CD4+ and CD8+ TRM differ in marker topology and function: CD8+ TRM more frequently co-express CD103, whereas CD4+ TRM are often CD103- but CD49a+/CXCR6+ and retain the plasticity to adopt Th1, Th17, Tfh, or Th2 fates dictated by local cytokines (11, 17). This heterogeneity is central to AR, because the nasal mucosa is a type-2-biased environment in which TRM may be instructed toward pathogenic Th2 programs (**Table 1**).

**Table 1:** Markers and functional attributes of nasal/airway TRM relevant to AR.

Marker / TRM subset	Function / property	Evidence in nasal / airway disease
CD69	Antagonizes S1PR1, retains T cells in tissue; canonical residency marker	Enriched in nasal polyps (CRSwNP) vs blood (16); upregulated on airway/lung TRM (4, 11, 18, 19)
CD103 (aEb7)	Binds epithelial E-cadherin; anchors CD8+ TRM at epithelium	Nasal CD8+ TRM more frequently CD103+ than CD4+ (16); barrier anchoring in mucosa (4)
CD49a (VLA-1)	Binds collagen; marks CD4+ TRM and cytolytic poise	Nasal CD4+ TRM are CD103- but CD49a+CXCR6+ in eosinophilic CRS (17)
CXCR6	Homing to CXCL16-rich niches; sustains residency	High CXCR6 on nasal CD4+ TRM in eosinophilic CRS (17)
Th2 program (GATA3/CRTH2) in CD4+ TRM	Type-2 cytokine output (IL-4/IL-5/IL-13)	Nasal CD4+ TRM Th2-polarized, correlated with severity (17); early IL-4+ nasal memory T cells (21)
GZMK / KLRG1 in CD8+ TRM	Cleaves complement C2-C5; drives recurrent inflammation	Clonally expanded GZMK+CD8+ TRM in recurrent airway disease (Nature 2025) (30)

Evidence in this table is drawn largely from CRSwNP and lower-airway studies [references (4, 11, 16–19, 30)]; direct demonstration of these markers and functions in the nasal mucosa of pure AR is still limited and should be regarded as inferred rather than established.

## 3 Human evidence for nasal TRM in allergic disease

The notion that the nasal mucosa harbors allergen-responsive memory T cells is not new. Already in 1992-1994, Hellquist and Karlsson reported CD4+CD45RO+ memory T cells in the nasal mucosa of allergic patients that expanded during pollen season and were capable of IL-4 production - an early hint of local, type-2-polarized memory (1, 21). What has changed is the resolution: flow-cytometry and single-cell studies now demonstrate genuine tissue residency.

In chronic rhinosinusitis with nasal polyps (CRSwNP), Ickrath and colleagues first documented accumulation of CD69+S1PR1- TRM (both CD4+ and CD8+) in polyp tissue relative to blood (16). More recently, Rha and colleagues profiled nasal CD69+CD4+ TRM in eosinophilic CRS, showing these cells are almost exclusive to nasal tissue, express CD49a and CXCR6, and contain a high-frequency Th2 (CRTH2+GATA3+) compartment with robust IL-4/IL-5 production - and their abundance correlates with disease severity and olfactory loss (17) (a single-center, modest-sample series that is indicative rather than definitive). Two contemporary reviews consolidate the field: Moon and Rha position nasal TRM, Tfh, and gd T cells as persistent drivers of CRS recurrence (22), while Koo and colleagues frame ‘mucosal inflammatory memory’ - encompassing pathogenic TRM/TRB cells, epithelial stem-cell imprinting, and trained innate immunity - as the basis for postoperative and post-biologic relapse (23). Whether gd T cells contribute a distinct memory circuit in the nasal mucosa of AR is, however, unexplored and beyond the present scope.

A parallel clinical observation reinforces the concept of a locally restricted nasal memory: local allergic rhinitis (LAR), in which patients show nasal allergic inflammation and positive nasal allergen challenges despite negative skin-prick tests and absent systemic specific IgE, points to a mucosa-bound memory state that systemic atopy assays miss (24, 25). LAR is therefore consistent with - and indirectly supports - a local nasal memory circuit, but it does not by itself demonstrate that TRM cells are the cellular driver; we treat it here as circumstantial rather than direct evidence. Notably, these advances have occurred almost entirely in CRS/CRSwNP. Dedicated human studies of nasal TRM in pure AR remain scarce - a gap this review seeks to highlight. The translational logic, however, is strong: AR and eosinophilic CRS share a type-2 mucosal milieu and the same nasal tissue niche, so mechanisms established in polyps likely operate, at lower intensity, in AR. Throughout, we flag conclusions that rest on CRS/airway data as ‘inferred’ for AR.

## 4 Cellular circuits: how nasal TRM sustain type-2 inflammation

Three interconnected circuits position nasal TRM as amplifiers of AR (**Figure 1**).

**Figure 1:**
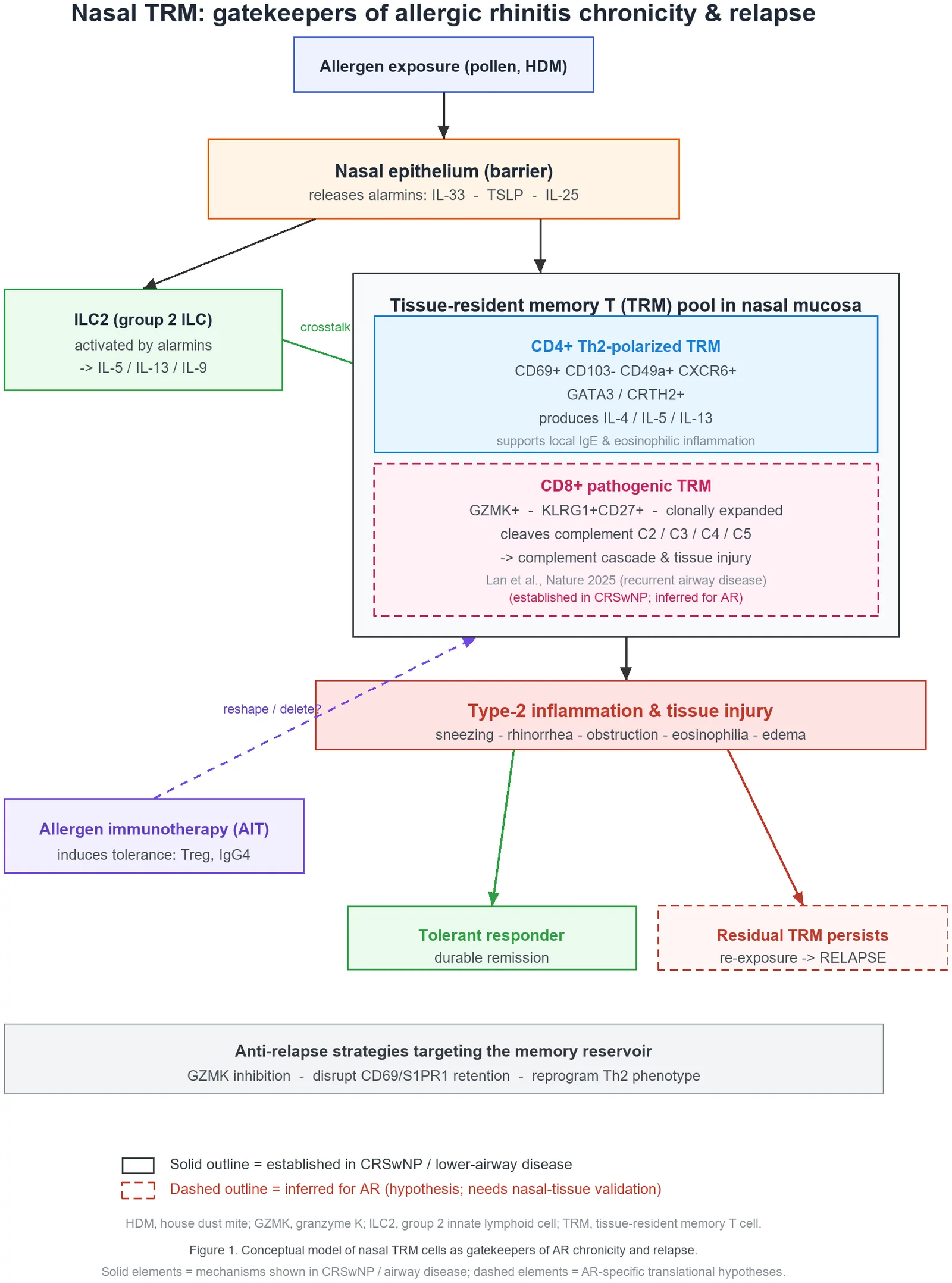
Conceptual model of nasal tissue-resident memory T (TRM) cells as gatekeepers of allergic rhinitis (AR) chronicity and relapse. Solid elements denote mechanisms established in chronic rhinosinusitis with nasal polyps (CRSwNP) or lower-airway disease and inferred - but not yet proven - for pure AR; dashed elements denote the AR-specific translational hypothesis. Allergen encounter at the nasal epithelium triggers release of epithelial alarmins (IL-33, TSLP, IL-25) that activate group 2 innate lymphoid cells (ILC2) and resident T cells (26–29). Two TRM arms operate: (i) CD4+ Th2-polarized TRM (CD69+CD103-CD49a+CXCR6+) that produce IL-4/IL-5/IL-13 and support local IgE and eosinophilic inflammation (17, 21); and (ii) a pathogenic CD8+ TRM subset (GZMK+, KLRG1+CD27+) that cleaves complement components to amplify tissue injury (30) - demonstrated in CRSwNP, inferred for AR. On discontinuation of allergen immunotherapy (AIT), a residual TRM pool persists and is reawakened by re-exposure, driving relapse (2, 3, 33); in tolerant responders, the local memory circuit is durably reshaped (23, 33). The persistence and reactivation of nasal TRM after AIT - and the GZMK+CD8+ arm in AR specifically - are hypotheses requiring direct nasal-tissue validation. TRM, tissue-resident memory T cell; GZMK, granzyme K.

First, local re-stimulation. On allergen re-entry, nasal TRM, poised at the epithelial interface, can be reactivated within hours, bypassing the days-long recruitment of circulating cells (4, 9). Th2-polarized CD4+ TRM directly secrete IL-4, IL-5, and IL-13, sustaining eosinophil survival, mucus hypersecretion, and the IgE-facilitating environment (demonstrated in eosinophilic CRS (17) and early nasal studies (21); inferred for pure AR).

Second, epithelium-TRM-ILC2 crosstalk. Barrier epithelial cells release alarmins (IL-33, TSLP, IL-25) upon allergen or protease exposure; these instruct group 2 innate lymphoid cells (ILC2) and can co-activate TRM at the mucosa (26–29). Resident TRM thus sit at the nexus of innate and adaptive type-2 signaling, converting a single environmental cue into a sustained adaptive cascade.

Third, the CD8+ TRM and the pathogenic memory loop. Long considered purely antiviral, nasal CD8+ TRM are increasingly implicated in chronic inflammation (16). The landmark 2025 Nature study by Lan, Zhang, Qi and colleagues redefined this arm: in patients with recurrent CRSwNP, persistent clonally expanded CD8+ memory T cells - marked by granzyme K (GZMK), with a KLRG1+CD27+ effector-memory-like phenotype - colonize nasal tissue, directly cleave complement components (C2, C3, C4, C5) to ignite the complement cascade, and continuously traffic from blood to tissue to drive flare-ups (30). GZMK tissue levels outperformed eosinophilia and IL-5 as predictors of severity and comorbidity (30). These mechanisms are firmly established in recurrent CRSwNP and a murine asthma model; their specific contribution to pure AR remains a testable hypothesis that awaits direct nasal-tissue validation.

## 5 Chronicity and relapse: a pathogenic-memory framework for AR

We propose that AR chronicity and post-AIT relapse can be unified by a TRM-centric model (**Figure 1**). In this model, each allergen season or AIT course leaves behind a residual nasal TRM pool - Th2-polarized CD4+ cells and, where present, GZMK+CD8+ cells - that is not erased by systemic treatment. On re-exposure, these cells are rapidly reawakened, re-establishing type-2 inflammation and, via the CD8+/complement axis, amplifying tissue injury. The framework is directly translatable from the CRS recurrence mechanism of Lan et al. (30) and the ‘mucosal inflammatory memory’ concept of Koo et al. (23); we stress that in AR it is a hypothesis to be tested, not an established fact. It explains why symptomatic control (antihistamines, intranasal steroids) and even antigen-specific tolerance (AIT) often fail to deliver cure: they may modulate the systemic or effector limb but leave the local memory reservoir intact.

A key corollary concerns AIT durability. AIT is uniquely disease-modifying, affording remission that outlasts treatment through induced tolerance (Treg, IgG4 blocking antibodies, Th2 to Th1 deviation) (2, 3, 31–33). Yet relapse after discontinuation is common, and current biomarkers cannot predict who will relapse (2). We hypothesize that the composition and durability of the nasal TRM pool - its Th2 content, its GZMK+CD8+ fraction, and its persistence after AIT - determines long-term outcome. If AIT reshapes rather than deletes nasal TRM, patients with residual pathogenic TRM would be precisely those who relapse. It is important to state this link explicitly as a hypothesis: the human evidence that AIT durably remodels nasal TRM is currently lacking. What is shown is that grass-pollen AIT induces Foxp3-expressing regulatory T cells in the nasal mucosa (33) - a local tolerogenic change, but one that does not by itself demonstrate deletion or phenotypic rewiring of TRM. The AIT-TRM relationship therefore remains to be established. This reframing nonetheless suggests that AIT efficacy should be read out not only in blood (IgG4, basophil suppression) but at the tissue level, by profiling nasal TRM before, during, and after treatment.

## 6 Therapeutic implications and open targets

If nasal TRM are gatekeepers of relapse, three intervention logics emerge. (i) Disrupt retention: agents that relieve CD69-mediated S1PR1 antagonism or block CD103/CD49a adhesion could destabilize residency and permit TRM egress and clearance (12, 13). (ii) Reprogram phenotype: cytokines or small molecules that divert nasal CD4+ TRM away from Th2 (e.g., reinforce Treg or Tr1 programs) could dampen local type-2 output without systemic immunosuppression. (iii) Neutralize effectors: the GZMK-complement axis offers a concrete, druggable target - Lan et al. showed that genetic or pharmacological GZMK inhibition, tested in cells derived from recurrent CRSwNP patients and in a murine asthma model, alleviated pathology and restored lung function (30). A GZMK inhibitor would be a prototype ‘anti-relapse’ drug acting on the memory reservoir rather than on symptoms; direct evidence in AR is, again, pending.

Each strategy must weigh benefit against the protective role of TRM against respiratory viruses - a safety consideration that will require tissue-specific delivery.

## 7 Outstanding questions

(a) What are the precise transcriptional and clonal dynamics of nasal TRM in pure AR versus CRS, and do seasonal and perennial AR differ? (b) Can GZMK+CD8+ pathogenic TRM be demonstrated in AR nasal tissue, not only in polyps? (c) Does AIT quantitatively or qualitatively remodel nasal TRM, and does post-AIT TRM composition predict relapse? (d) Are nasal TRM organized in tertiary lymphoid structures that seed their maintenance, as suggested for CRS (22, 30, 34)? (e) Which TRM-associated molecules are both effective and safe targets given the antiviral functions of mucosal TRM?

## 8 Conclusion

Nasal TRM cells are positioned at the intersection of allergen exposure, type-2 inflammation, and durable immune memory. The strongest human evidence comes from CRSwNP - capped by the 2025 discovery of GZMK+CD8+ pathogenic memory T cells in recurrent airway disease (30) - and we have argued that this evidence is translatable, though not yet proven, to AR. The model in which nasal TRM gate the chronicity and relapse that define the AR clinical experience is therefore a reasoned synthesis and a testable hypothesis rather than an established doctrine. Centering TRM in AR biology reframes relapse not as treatment failure but as the persistence of a local memory circuit, and it opens concrete paths - from retention blockers to GZMK inhibition - toward true disease modification that remain to be validated in dedicated AR cohorts. We note, finally, that nasal TRM are likely one component of a broader mucosal immune-memory network - encompassing epithelial imprinting, trained innate immunity, and B-cell memory - rather than necessarily the sole cellular reservoir of disease persistence; the TRM-centric view should therefore complement, not replace, the multifactorial understanding of AR chronicity.
